# Association between organizational citizenship behavior and patient safety culture from nurses’ perspectives: a descriptive correlational study

**DOI:** 10.1186/s12912-020-00416-y

**Published:** 2020-04-14

**Authors:** Marzyeh Jafarpanah, Behrooz Rezaei

**Affiliations:** 1 Department of nursing, Nursing & Midwifery Faculty, Isfahan (Khorasgan) Branch, Islamic Azad University, Isfahan, Iran; 2 Nursing & Midwifery Faculty, Falavarjan Branch, Islamic Azad University, Isfahan, Iran

**Keywords:** Organizational culture, Patient safety, Hospital survey on patient safety culture (HSOPSC), Safety management, Cross-sectional studies, Hospitals, Teaching

## Abstract

**Background:**

Nurses play a key role in providing patient safety. It is known that patient safety requires the improvement of patient safety culture, which can be a difficult process. One of the current challenges of hospitals is to explore the ways to improve patient safety culture. Organizational citizenship behaviors are one of the factors, which can develop organizational culture including safety culture; however, its role is not well established.

**Methods:**

In this cross-sectional study, a stratified random sample of 214 nurses was selected from a largest teaching hospital in west of Iran. The institutional research board approved the study protocol. Data were collected using three self-report questionnaires: demographic information; hospital survey on patient safety culture (HSPSC); and organizational citizenship behaviors questionnaire. Data were analyzed using Spearman’s correlation coefficient test in SPSS (α < 0.05).

**Results:**

Organizational citizenship behaviors were found to be at an intermediate level (56.84 ± 16.22). However, some of its dimensions, including sportsmanship, civic virtue, and courtesy, were at weak levels (< 50%). The mean percentage of positive responses to the patient safety culture was 49.00 ± 14.01. The patient safety culture had significant positive correlations with organizational citizenship behaviors (*r* = 0.349, *P* = 0.001) and dimensions of altruism (*r* = 0.255, *P* = 0.001), civic virtue (*r* = 0.434, *P* = 0.001), and courtesy (*r* = 0.214, *P* = 0.001).

**Conclusion:**

Our findings proposed the hypothesis that OCB has a statistical significant impact on PSC. Low levels of civic virtue, sportsmanship and courtesy behaviors may be indicative low nurses’ interest in participating in organizational affairs and nurses’ low attention to measures that prevent harm to their organization. It is recommended that nursing managers focus more on these dimensions, identifying influintioal factors and taking appropriate management measures to promote these behaviors. If our findings are confirmed in future studies, nursing managers can consider the development of organizational citizenship behaviors as one of the managerial approaches for promoting a patient safety culture.

## Background

Patient safety is a vital part of healthcare quality [1]. Today, healthcare organizations focus on the development of Patient Safety Culture (PSC), and formation of safety culture is dependent on the better performance of these organizations [2]. PSC includes common values and ideas, which create behavioral standards within an organization through interactions with organizational structures and control systems [1–3]. PSC is rooted in open communication, leadership, and teamwork ability of individuals, leading to their commitment to patient safety [2]. Development and maintenance of PSC can lead to the better performance of healthcare providers [2, 3].

Among health professions, nurses play a key role in ensuring the quality and safety of healthcare services [1]. Recent studies have shown that high levels of organizational commitment [3, 4] and job satisfaction [5] of nurses have positive effects on PSC. In addition, a PSC has positive effects on the nurses’ performance [4] and hospital efficiency [2]. Therefore, measurement of PSC provides the necessary information for promoting patient safety and helps monitoring the changes needed at any time [5, 6].

On the other hand, the concept of organizational citizenship behavior (OCB) has been considered in recent studies [7–15].OCB typically includes optional behaviors beyond the formal roles of employees, which are not directly recognized by the organizational reward system [16]. Organ (1988) identified five dimensions of OCB, including civic virtue, altruism, conscientiousness, sportsmanship, and courtesy. Civic virtue, altruism, and conscientiousness are regarded as positive and useful factors, while sportsmanship and courtesy are components, which represent avoidance of damage to the organization [12]. Indeed OCB refers to maintaining and enhancing social and psychological contexts, which support task performance. This definition emphasizes on three characteristics of these behaviors: voluntariness, organizational benefits, and multifaceted nature [7]. In the other, OCB also includes three main parts: employees’ personal traits, employees’ attitudes, and managerial factors. Employees’ attitudes and managerial factors are modifiable, while employees’ personal traits are the least flexible. The most important of these three factors is the role of management, as it can promote healthy and constructive behaviors among employees [7, 11]. OCB improves employees’ participation, encourages teamwork, reduces error costs, and provides a positive organizational environment [11, 13]. It also decreases the need for staffs’ monitoring, and exerts positive effects on organizational performance and providing high-quality services [8, 13].

Previous studies have shown that management practices have a significant positive effect on OCB and that OCB can be strengthened by appropriate leadership styles and management policies [7, 17–21]. In this regard, Ozturk and Ay [18], and Rogers [20] showed that leadership, especially transformational leadership, has a positive effect on OCB and employees’ commitment. A number of studies have shown that spirituality in the workplace [11, 14], emotional intelligence [9], job satisfaction [8], and social capital [10] are effective on nurses’ OCB. In return, improving nurses’ OCB seems to influence the safety climate [13], job performance [15] and quality of healthcare services [8].

In the other hand, PSC emphasizes on the formation of a system, which improves prediction and prevention of errors, and promotes patient safety and health care quality [22, 23]. Organizations with a positive PSC are established based on mutual trust-based communication and are recognized through effective preventive measures and have a higher safety function and fewer adverse events [23, 24]. Since healthcare organizations strive to improve the quality of care, current knowledge on the importance of safety culture has increased [22]. Nursing managers need tools which can help them in order to develop PSC [25]; therefore, more efforts must be made at organizational levels. Meanwhile, the first step in interventions is measuring PSC and related factors, which can give an insight into all dimensions [2]. Although numerous studies have been conducted on commercial and industrial organizations, research on health organizations is still insufficient.

### Healthcare system and nursing profession in Iran

Iran’s health system includes a big public sector, which is complemented by the private sector [26]. The Ministry of Health runs a comprehensive healthcare network that provides primary, secondary, and tertiary healthcare services, with advanced services provided in a variety of hospitals including public, private, or semi-private [26, 27]. In the past three decades, Iran’s Health System has progressed, leading to the improvement of Iranians’ health status [26]. Professional nursing has also progressed substantially in recent years. The majority of healthcare services are provided by teaching hospitals, and most nurses working in these hospitals have a bachelor’s degree.

However, Iran’s health system faces a number of important challenges, especially regarding the quality of healthcare services and health performance [26]. There are also serious challenges in nursing profession, such as job dissatisfaction, poor social status, nursing shortage, heavy workload and gap between theory and practice. Evidence suggests that Iranian nurses experience higher levels of occupational stress, workload, and burnout in comparison with nurses in other countries; this reduces the quality of nursing care and leads to patient dissatisfaction [27, 28]. Despite advances in nurses’ educational level, people and government criticize the low quality and safety of care [29]. It should be noted that changing PSC is difficult and requires multilateral management measures. Considering the current status of Iran’s health system, determining the effects of nurses’ OCB on PSC can provide a new approach to the development of PSC for hospitals managers.

### Conceptual framework and aim of the study

Since organizations are increasingly faced with external economic pressures to use more limited resources, OCB can be a vital factor in organizational success [13]. In addition, OCB plays a significant role in providing high-quality services to internal and external customers [8, 13]. In previous studies have shown that management practices have a significant positive effect on OCB [7, 17–21] and that OCB can be strengthened by appropriate leadership styles and management policies [18, 20].

On the other hand, one of the essential requirements of health organizations is to ensure patient safety, which requires making and developing a positive patient safety culture, but experiences show that improving PSC can be difficult. One of the current challenges for hospital managers is exploring the ways to improve patient safety culture. OCB is one of the factors that can develop organizational culture. According to the current literature, we can develop OCB through managerial policies and leadership styles. Since organizational culture influences the safety culture, it seems that development of OCB can lead to improved PSC.

Patient safety culture and organizational citizenship behaviors are both aspects of organizational culture in health care organizations that appear to be related to each other and may influence each other, but so far this relationship has not been explored. To the best of our knowledge, this is the first attempt to investigate the relationship between OCB and PSC. Therefore, the main objectives of this study are:

- Exploring the status of OCB from the nurses’ perspective.
- Exploring the relationship between OCB and PSC from the nurses’ perspective.

## Methods

### Study design& setting

This was a cross sectional study using descriptive-correlational design to investigate the association between the OCB and PSC from nurses’ perspective. The study was conducted in the largest teaching hospital in west of Iran (Kermansha province) during December 2017.

### Participants & sample

The statistical population consisted of all registered nurses (*N* = 470) working in medical surgical wards of the largest teaching hospital in west of Iran. The sample size was determined to be 214 based on the Krejcie and Morgan’s table. In fact, if the total population is known, the simplest way to determine the sample size is to use the Morgan table [30]. The subjects were selected through stratified random sampling method. In this method, participants are divided into various sub-groups (strata) sharing common characteristics like age, sex, race, income, education, ethnicity and workplace. A random sample is taken from each stratum. Therefore in sampling procedure, first nurses is divided into various sub-groups (strata) sharing common nurses’ workplace (clinical wards). Then the samples were selected by simple random sampling in each hospital ward.

Permission was first obtained from the ethics committee and hospital management board, and then, based on the inclusion criteria, questionnaires were distributed among nurses, who had given their informed consents to participate in the study. The nurses were asked to complete the questionnaires on the same day and return them to the researchers. Finally, according to the exclusion criteria, questionnaires of 203 participants were analyzed (response rate = 94.85%). The inclusion criteria of the study were as follows: having clinical work experience for six months or longer; having a bachelor’s degree or higher in nursing; and giving an informed consent for participation. The questionnaires, which were not filled completely, were excluded from the study.

### Measures

Data were collected using two questionnaires. The questionnaires were written and self-reported. In addition, demographic information (e.g., gender, age, marital status, education level, work experience, and job category) was gathered. The first questionnaire was the Persian version of the OCB questionnaire [8, 31]. The original version of this questionnaire was designed by Kanovsky and Organ (1996) and contains 15 items [32]. It has been used internationally to measure OCB from the employees’ perspective. The items of this questionnaire are scored based on a five-point Likert scale (from strongly disagree =1 to strongly agree = 5). The options “strongly agree” and “agree” are considered as positive responses, while “strongly disagree” and “disagree” are considered as negative responses. In order to determine the score for each dimension, the total score of positive responses related to that dimension was calculated on a scale of 100. Overall, the mean percentage of positive responses below 50% indicates a weak level of OCB, which needs to be promoted. In order to measure the total score of the questionnaire, the scores of all items were summed [31].

The OCB questionnaire includes five subscales of altruism (3 items), conscientiousness (3 items), courtesy (2 items), civic virtue (3 items), and sportsmanship (4 items). Civic virtue is characterized by behaviors that show nurses’ deep concerns, active interest, and participation in the organization’s life [12, 18]. Conscientiousness is a behavior beyond the defined organizational requirements (e.g., using time more efficiently or producing at higher levels). Altruism is to volunteer help to fellow colleagues, leading to good professional relations. Sportsmanship refers to enduring inevitable complications and overworking without objection. Finally, courtesy reflects the way nurses interact with colleagues, supervisors, and clients of the organization [16, 18]. The validity of the Persian version of this questionnaire has been confirmed using the content validity method [8, 31]. Its reliability has been also confirmed based on the test-retest method with a Cronbach’s alpha coefficient of 0.70–0.85 in national studies [8, 31].

The second questionnaire was the Persian version of Hospital Survey on Patient Safety Culture (HSOPS) [33, 34]. The original version of the HSOPS was designed by the Agency for Healthcare Research and Quality (AHRQ). HSOPS is a valid and reliable psychometric tool for assessing PSC from the hospital staff’s perspective [22, 23, 35–37]. It includes 12 subscales (manager expectations and actions promoting patient safety; organizational learning; teamwork within units; communication openness; feedback and communication about errors; non-punitive response to errors; staffing; management support for patient safety; teamwork across units; handoffs and transitions; overall perceptions of safety; and frequency of events reported) and 42 items. Each subscale consists of three to five items, rated on a five-point Likert scale, ranging from strongly agree (always) to strongly disagree (never). The total score of the tool ranges from 42 to 210 [36–38]. Various studies have reported the adequate reliability and validity of HSOPSC worldwide [22, 37–39]. Validity and reliability of the Persian version of HSOPSC have been confirmed, using confirmatory factor analysis, internal consistency, and test-retest [33].

### Data analysis

Data were analyzed using descriptive and analytical tests in SPSS version 17. All tests were two-tailed, and the significance level was set at 0.05. For descriptive variables, statistical indices, such as frequency, percentage, mean (average), and standard deviation (SD), were measured to assess the nurses’ demographic information and scores of HSOPS and OCB questionnaires. Kolmogorov-Smirnov test and Spearman’s correlation coefficient test were used as analytical tests. Kolmogorov-Smirnov test showed that the scores of HSOPS and OCB did not have normal distribution. Since in the initial analysis, the data did not meet some conditions of the linear regression model (normal distribution, normal residuals or errors, homoscedasticity, uncorrelated errors, and zero conditional mean error), regression was not considered to examine the association between OCB and PSC. “Linear regression is a powerful statistical model when used correctly. Because the model is an approximation of the long-term sequence of any event, it requires assumptions to be made about the data it represents in order to remain appropriate. However, these assumptions are often misunderstood”. This issue can produce misleading conclusions [40]. Therefore, Spearman’s correlation coefficient was performed to investigate the relationship between OCB and PSC.

### Ethical considerations

The ethical considerations of the study included anonymity of the questionnaires and maintaining the confidentiality of information. In addition, approval was obtained from the ethics committee and hospital management board, and informed consents were collected from the participants. The questionnaires were used after obtaining the required permissions.

## Results

The results showed that the total score of OCB was at a moderate level. In addition, the results showed a significant positive correlation between OCB and PSC. Although this correlation was not strong, it was statistically significant.

### Response rate and respondents’ characteristics

In this study, 203 nurses completed the questionnaires and returned them to the researchers (response rate = 94.9%). The majority of the participants (78.8%) were female, and more than half of them (52.7%) were single. In addition, 87.7% of the participants had a bachelor’s degree of nursing (Table 1). The average age and working experience of nurses were 31.64 ± 6.05 and 5.68 ± 3.59 years, respectively. Also, the nurses’ average work time was 48.53 ± 7.66 h per week (Table 1).

**Table 1:** Demographic information of nurses studied working in medical surgical wards

Variables	Categories	N (%)
**Gender**	Male	43 (21.2)
	Female	160 (78.8)
**Marital status**	Single	107 (52.7)
	Married	96 (47.3)
**Age (years)**	< 30	93 (45.8)
	31–40	91 (44.8)
	> 40	19 (9.4)
**Education level**	Bachelor’s degree	178 (87.7)
	Master’s degree	25 (12.3)
**Work experience (years)**	< 5	100 (49.3)
	5–10	95 (46.8)
	> 10	8 (3.9)
**Job category**	Managerial (Head nurse, Supervisor)	16 (7.9)
	Non managerial (Staff/ Clinical Nurse)	187 (92.1)

### OCB and PSC scores

Regarding the nurses’ OCB, the mean percentage of positive responses below 50%, 50–75%, and 75–100% was considered to be weak, intermediate, and good, respectively. The results showed that the mean percentage of positive responses regarding OCB was 56.84 ± 16.22, which was at an intermediate level. Moreover, the mean percentage of positive responses to the dimensions of conscientiousness and altruism was higher than 75%, which was evaluated to be good. However, regarding the dimensions of sportsmanship, civic virtue, and courtesy, the mean percentage of positive responses was below 50%, which was considered to be weak. The courtesy dimension showed the lowest average percentage of positive responses (Table 2). In addition, the mean percentage of positive responses to the patient safety culture was 49.00 ± 14.01.

**Table 2:** Mean and standard deviation of percent of positive responses in all dimensions of OCB* scale

Dimensions	Number of items	Mean	SD**	Rank
**Conscientiousness**	3	86.5	23.3	1
**Altruism**	3	76.0	25.4	2
**Sportsmanship**	4	46.8	26.5	3
**Civic virtue**	3	43.2	26.1	4
**Courtesy**	2	38.3	23.6	5
**OCB** (Total)	15	56.8	16.2	–

*OCB Organizational citizenship behavior

**SD Standard deviation

## Relationship between OCB dimensions and PSC

The results of Kolmogorov-Smirnov test showed that the scores of OCB (z = 0.11, *P* = 0.001) and PSC (z = 0.07, *P* = 0.032) did not have a normal distribution. Spearman’s correlation coefficient test showed a significant positive correlation between OCB and PSC (*r* = 0.35, *P* = 0.001). Furthermore, the scores of altruism (*r* = 0.26, *P* = 0.001), civic virtue (*r* = 0.43, *P* = 0.001), and courtesy (*r* = 0.21, *P* = 0.001) dimensions had significant positive correlations with PSC. Although these correlations were weak, they are statistically significant (Table 3).

**Table 3:** Spearman correlation coefficient (r) between dimensions of OCB and PSC

Item	1	2	3	4	5	6	7
1. Altruism	1						
2. Conscientiousness	0.47^c^	1					
3. Sportsmanship	0.10	0.19^b^	1				
4. Civic virtue	0.17^a^	0.08	0.07	1			
5. Courtesy	0.16^a^	0.02	−0.18^a^	0.52^c^	1		
6. OCB overall	0.63^c^	0.58^c^	0.37^c^	0.64^c^	0.56^c^	1	
7. PSC overall	0.247^c^	0.090	0.084	0.295^c^	0.309^c^	0.401^c^	1

*PSC* Patient safety culture

*OCB* Organizational citizenship behavior

^a^Correlation is significant at the 0.05 level (2-tailed)

^b^Correlation is significant at the 0.01 level (2-tailed)

^c^Correlation is significant at the 0.001 level (2-tailed)

## Discussion

The main goal of this study was to examine the status of OCB and its relationship with PSC from the nurses’ point of view. Our findings indicated that OCB and PSC were at moderate levels from the nurses’ viewpoint. However, regarding the nurses’ OCB in dimensions of courtesy, civic virtue, and sportsmanship, the mean percentage of positive responses was below 50% and at a poor level. Similarly, Yaghoubi et al. [31] Yu et al. [41] showed that the nurses’ OCB was reported to be at a moderate level. Nevertheless, Fathizadeh et al. [8], Taghinezhad et al. [11], and Clark et al. [13] reported that the nurses’ OCB was satisfactory. The discrepancy between the findings is probably due to differences in personal and organizational factors affecting OCB. Based on past research, OCB leads to better individual and organizational performance [7, 8, 10, 11, 13, 15, 17, 41] and these behaviors can be enhanced through managerial measures, especially appropriate leadership style [18, 20]. Therefore, considering that nurses are the largest healthcare staffs who are responsible for quality health care in hospitals, assessing the status of these behaviors in nurses is one of the priorities of nursing management. Evaluating the level of organizational citizenship behaviors of nurses will help nursing managers to formulate plans and take management actions to improve these behaviors.

Our study indicated that the nurses’ OCB in dimensions of sportsmanship, civic virtue, and courtesy were at weak level. Similarly, Yaghoubi et al. [31] and Taghinezhad et al. [11] showed that the OCB of Iranian nurses in dimensions of courtesy and civil virtue had the lowest positive scores [11, 31]. However, Yu et al. showed that the OCB of male nurses in dimensions of civic virtue and sportsmanship had the highest scores [41]. The discrepancy between the findings may be attributed to differences in nationality, personal characteristics, work environment, and different organizational factors affecting organizational behaviors. Since the sportsmanship and courtesy are components, which represent avoidance of damage to the organization [8, 11, 16, 33], low scores on these domains may be indicative of the nurses’ low attention to measures that prevent harm to their organization. In addition civic virtue is known as behaviors that reflect attention to social participation in organizational life [11, 16, 17, 31], therefore, low levels of civic virtue behaviors may indicate low nurses’ interest in participating in organizational affairs. It is recommended that nursing managers focus more on these dimensions, identify the factors that decrease these behaviors, and take appropriate management measures to promote this group of organizational citizenship behaviors.

Another objective of this study was to examine the relationship between OCB and PSC. Our findings showed that OCB (total score) and its dimensions, including altruism, civic virtue, and courtesy, had significant positive correlations with PSC. Although these correlations were not strong, they are statistically significant. To the best of our knowledge, there is no similar study in the literature for comparing this finding.

Given the weakness of the reported correlation coefficient and the limitations of present study (conducting a study at a health institution, and cross-sectional and correlational design) this finding may not significant. But it can contribute to the development of future studies in this field. Future studies with larger sample size at multiple institutes are recommended to confirm the findings of this study. If this finding is confirmed in future studies, nursing managers can consider the development of organizational citizenship behaviors as one of the managerial approaches for promoting patient safety culture.

### Limitations of the study

Novelty and high response rate are the strengths of this study. However, conducting study in one healthcare organization, study design and the statistical analysis (due to abnormal data distribution) are the limitations of this study, which affect the generalizability of our results.

## Conclusion

According to the present study, improving nurses’ OCB, especially in dimensions of sportsmanship, civic virtue, and courtesy through managerial practices is suggested. Low levels of civic virtue, sportsmanship and courtesy behaviors may be indicative low nurses’ interest in participating in organizational affairs and nurses’ low attention to measures that prevent harm to their organization. Our findings proposed the hypothesis that OCB has a statistical significant impact on PSC although it needs to be confirmed in future studies. Our study contributes to the development of research in this field. In healthcare organizations, formation and development of PSC are critical to patient safety and care quality. Therefore, identifying the factors influencing the safety culture is one of the nursing managers’ responsibilities, and nurses’ OCB is an important factor, which has not been well recognized in hospitals.

Although this is a small-scale study, the results may be used to similar hospitals in similar contexts. If our findings are confirmed in future studies, nursing managers can consider the development of organizational citizenship behaviors as one of the managerial approaches for promoting patient safety culture. Nursing managers often face major problems in the promotion of safety culture and quality of healthcare services. According to previous studies, it is clear that PSC is effective in providing safe and high-quality care. However, it should be noted that changing PSC is difficult and requires multilateral management measures. Therefore, determining the effect of nurses’ organizational citizenship behavior on the safety culture can provide a new approach to the development of safety culture for nursing managers. We recommend that future studies use a larger sample size in hospitals of other countries.

## Data Availability

The datasets used in the present study are accessible from the corresponding author on reasonable request.

## Abbreviations

AHRQ: Agency for healthcare research and quality
HSOPSC: Hospital survey on patient safety culture
OCB: Organizational citizenship behavior
PSC: Patient safety culture
